# Psychometric Testing of an Indonesian-Version Diabetes Self-Management Instrument

**DOI:** 10.1097/jnr.0000000000000403

**Published:** 2020-10-08

**Authors:** Henik Tri RAHAYU, Ching-Min CHEN

**Affiliations:** 1 MSN, Doctoral Student, International Doctoral Program in Nursing, College of Medicine, National Cheng Kung University, Taiwan, ROC, and Lecturer, Department of Nursing, Health Sciences Faculty, University of Muhammadiyah Malang, Malang, Indonesia; 2 RN, DNS, Professor, Department of Nursing, Institute of Gerontology, and Institute of Allied Health Sciences, National Cheng Kung University, Taiwan, ROC.

**Keywords:** instrument development and validation, self-management, diabetes, primary healthcare

## Abstract

**Background:**

Self-management is one of the vital elements in diabetes management for adults with Type 2 diabetes mellitus (T2DM). Although the number of people with T2DM in Indonesia has risen, clinical understanding of the problems related to practicing diabetes self-management (DSM) is limited because of the lack of a valid measurement instrument. The 35-item Diabetes Self-Management Instrument (DSMI-35) is one instrument widely used in research to assess DSM-related behavior among patients with diabetes.

**Purpose:**

This study was designed to translate the psychometric properties of the Indonesian version of the DSMI-35 and evaluate the efficacy of this instrument in a sample of Indonesian adults with T2DM.

**Methods:**

Forward and backward translation processes were used to translate the DSMI-35 into Indonesian (IDN-DSMI). Then, the translation equivalence, content validity, face validity, construct validity, and internal consistency were assessed using a sample of 222 Indonesian adults with T2DM from eight public health centers. Confirmatory factor analysis was used to test the data.

**Results:**

The confirmatory factor analysis confirmed that the 35 items all had acceptable goodness of fit. Although the analysis supported removing several of the items, removal of these items was not theoretically justified. The average variance extracted was acceptable, and composite reliability was satisfied. The Cronbach's alpha was .96 for the IDN-DSMI and .84–.93 for the subscales. The significant interitem correlations between some items were consistent with the findings of other previous studies.

**Conclusions/Implications for Practice:**

The IDN-DSMI is a valid and reliable instrument that may be used to measure DSM behavior in Indonesian patients with T2DM in primary healthcare settings.

## Introduction

The number of people with diabetes has been rising rapidly worldwide. In 2019, approximately 463 million adults were living with diabetes, with this number expected to rise to 578 million by 2030 and 700 million by 2045 (International Diabetes Federation, 2019). The rising prevalence of diabetes is associated with the escalating prevalence of obesity, which is a major diabetes risk factor. The global age-standardized prevalence of obesity among adults (aged 18 years and older) has increased 150% since 2016 (World Health Organization, 2020b). Moreover, Indonesia, a developing country in the West Pacific region, had the seventh-largest population of people with diabetes in 2019. Indonesia is expected have the eighth-largest population of people with diabetes in 2045, with the country's 10.7 million people with diabetes in 2019 projected to grow to 13.7 million in 2030 and 16.6 million in 2045 (International Diabetes Federation, 2019).

Diabetes is currently one of the top noncommunicable disease (NCD) causes of death worldwide (World Health Organization, 2020b). In 2016, an estimated 41 million people worldwide (approximately 71% of total deaths) were attributable to NCDs, with approximately 1.6 million directly attributable to diabetes, making diabetes the fourth-largest NCD cause of death after cardiovascular disease, cancer, and chronic respiratory disease (World Health Organization, 2020b). Moreover, diabetes has been associated with a 5% increase in premature mortality. In Indonesia, diabetes is the third-largest direct cause of death after stroke and cardiovascular disease and was also identified as the largest burden disease in 2012 because of its high disability-adjusted life years (DALYs; Kementerian Kesehatan Indonesia, 2015). DALYs is a score equal to the sum of the number of years of life lost because of premature mortality and the number of years of healthy life lost because of disability (World Health Organization, 2020a).

The high DALYs associated with diabetes is believed to result from severe complications because of poor disease management. The long-term complications of diabetes may lead to heart disease, stroke, kidney disease, blindness, and amputation (Chamberlain et al., 2016). Diabetes and its complication are not only a health problem but also economic, social, and psychological burdens. This disease affects not only the individual but also families, health systems, and the entire country. The global health spending on diabetes treatment and related complication prevention was estimated to be at least USD 760 billion in 2019, which represents about 10% of total health expenditures on adults (International Diabetes Federation, 2019). Although no official information on diabetes expenditures in Indonesia, the International Diabetes Federation reported that total expenditures on diabetes in the Western Pacific region reached USD 162.6 billion in 2019 and are expected to rise to 184.7 billion in 2045 (International Diabetes Federation, 2019). Therefore, promoting disease management to control diabetes is an important strategy for reducing the risk of related complications and the cost for treatments.

### Background

Diabetes self-management (DSM) describes how people with diabetes practice self-care. DSM involves a patient's knowledge, attitude, and behavior to both maintain personal health and prevent long-term diabetes complications (International Diabetes Federation, 2012), with knowledge and attitude relating to the activities of daily living that a patient uses to stay healthy (Tol et al., 2011). The key elements of diabetes management are maintaining blood glucose level through dietary management, maintaining good exercise habits, taking prescribed medication, and monitoring blood glucose level to keep this level below 200 mg/dl and glycated hemoglobin A1c (HbA1c) at or below 7 (International Diabetes Federation, 2012). Moreover, on the basis of the American Association of Diabetes Educators, DSM consists of seven domains of self-management behaviors, including healthy eating, controlling blood glucose level, being active, taking medication, maintaining problem-solving abilities, reducing the risk of long-term complications, and having a healthy coping strategy for stress (American Association of Diabetes Educators, 2014). However, most patients with diabetes face obstacles in promoting self-management such as difficulties in coping with diabetes, self-monitoring, and lifestyle changes (Fidan et al., 2020). To evaluate the DSM compliance of patients, a reliable and valid tool to measure the quality of self-management behavior is necessary. However, there remains in Indonesia a widespread lack of information regarding DSM as well as a lack of valid, appropriate tools for assessing DSM status that are adaptable to individual conditions and assess the process rather than the outcome, allowing healthcare providers to identify problems in DSM practices.

Many instruments have been developed to measure DSM efficacy (Lu et al., 2016). Some measure DSM using patients' compliance or adherence to recommended activities to control blood glucose and prevent complications from diabetes. Some measures, including the Summary of Diabetes Self-Care Activities (Choi et al., 2016; Toobert et al., 2000) and Diabetes Self-Management Questionnaires (Schmitt et al., 2013), are based on the scope of the definition of “self-care” and “self-management” and measure how often adults with diabetes follow each recommended activity associated with controlling blood glucose level and reducing the risk of complications.

However, adults with diabetes have autonomy to manage their diabetes independent from healthcare professionals (Lin et al., 2008), and compelled compliance with a healthcare professional's advice may violate patients' value and autonomy (Anderson et al., 2000; Redman, 2009). To optimize quality of life, DSM should be flexible and adapted to individual conditions (Funnell & Anderson, 2004). Therefore, a preferred definition of DSM is “an active, flexible process in which patients develop strategies for achieving desired goals by regulating their actions, collaborating with their healthcare providers and significant others and performing preventive and therapeutic health-related activities” (Lin et al., 2008, p. 371).

The Diabetes Self-Management Instrument (DSMI), developed by Lin et al. (2008), is the only scale that measures DSM as a process evaluation rather than an outcome. The original instrument was developed in English, translated into Chinese, and then validated in Taiwan. The validation of the Chinese version showed appropriate content validity, internal consistency, and test–retest reliability. Farsi (Persian) and Vietnamese versions of the DSMI have also been translated and validated (Tahmasebi & Noroozi, 2012).

The 35-item, self-report DSMI is designed to assess the frequency with which adults with diabetes performed certain activities during the previous 3-month period using a 4-point Likert scale, with responses ranging from 1 (*never*) to 4 (*always*). The total score for the instrument ranges from 35 to 140, with higher scores representing a higher frequency of self-management activities. The DSMI incorporates the five subscales of self-integration (10 items), self-regulation (nine items), interaction with health professional and significant others (nine items), self-monitoring blood glucose (four items), and adherence to the recommended therapy (three items; Lin et al., 2008).

The validation of the Chinese version of this instrument on 634 adults with Type 2 diabetes mellitus (T2DM) in Taiwan achieved a Cronbach's alpha coefficient of .94 and a test–retest correlation of .73 (Lin et al., 2008). The Iranian version achieved an internal consistency of .91 overall and between .79 and .92 for each subscale as well as a test–retest correlation of .91 (Tol et al., 2011). The Vietnamese version earned an internal consistency of .91 overall and between .81 and .95 for each subscale (Dao-Tran et al., 2017).

### Aim

In this study, the original DSMI (35 items) was translated into Indonesian and its psychometric properties were tested to determine the acceptability and appropriateness of applying the translated version (IDN-DSMI) in populations of Indonesian adults with diabetes. It was expected that using the IDN-DSMI would give health professionals in Indonesia a better understanding of how Indonesian adults with diabetes self-manage their health and facilitate the design of appropriate DSM support for patients to reduce the risk of diabetes complications and improve overall health.

The purpose of this study was to conduct a psychometric test of the IDN-DSMI using confirmatory factor analysis (CFA).

## Methods

### Study Design

This study applied a quantitative study design using a cross-sectional survey. The research reporting guidelines were followed using the TRIPOD Checklist.

#### Phase 1: Development of the Indonesian version of the Diabetes Self-Management Instrument

First of all, permission to use the original instrument (Lin et al., 2008) was obtained from the original author. The DMSI was then translated into Bahasa (Indonesian) using a forward and backward translation process (Cha et al., 2007) to confirm linguistic equivalence. The English version was translated into Indonesian by two independent bilinguals (Indonesian–English) translators who were nurse lecturers. After the independent, forward English–Indonesian translation was completed, the research team held a consensus meeting with the two translators to establish a single translated version. Subsequently, the back-translated versions were compared with the original instrument by outside experts from the Language Center of Muhammadiyah University of Malang to identify any discrepancies.

After completing the forward and backward translation process, the research team conducted a content validity check of the IND-DSMI. Eight clinical and academic experts in diabetes care in Indonesia were asked to review the content validity of the instrument. An experienced endocrine physician, a medical–surgical nurse specialist, a nurse practitioner, and five lecturers on medical–surgical nursing at a nursing school participated in this content review, rating items on a scale of 1–4 (1 = *not relevant* and 4 = *very relevant*). The experts were further asked regarding the need to modify or eliminate each item. This study earned content validity ratio scores ranging from 0.5 to 1, with a mean score of .93, indicating that most questions are “essential.” The reviewer's comments focused primarily on changing word usage to clarify meanings. No reviewer suggested deleting any item. Finally, five patients with diabetes in the Indonesian community were invited to evaluate the face validity of the instrument and to assess from their individual perspectives the clarity of the instrument, ease of item understanding, ease of response, and fit with the purpose of the study (Yasir, 2016).

As mentioned above, adults with diabetes have autonomy to manage their diabetes independent of health professionals (Lin et al., 2008). Thus, although the focus group of patients would have been capable of confirming the cultural adaption and evaluating the content validity, these aspects were not addressed because of time and resource constraints. This condition is recognized as a limitation of this study. However, the original instrument was developed and validated in an Asian country with a culture similar to Indonesia's. Therefore, it hoped that the instrument is also valid for use in Indonesian settings.

#### Phase 2: Psychometric testing of the Indonesian version of the Diabetes Self-Management Instrument

A cross-sectional survey was used to test the IDN-DSMI to assess its validity and internal consistency.

##### Setting

Data were collected from July to September 2013 in eight endocrine outpatient departments of public health centers (PHCs) in Malang, Indonesia, using quota sampling methods based on the average daily patient visits to each PHC to calculate the proportion of the sample to be recruited from each PHC. Malang, the second-largest city in East Java, is home to the most people in East Java (3,266,461 people or 8.7% from the total population in East Java; Badan Pusat Statistik, 2010). The sample in this study was recruited from both urban and rural areas. The outpatient department at the PHCs were open 7:30 a.m. to 12:00 p.m. on Mondays through Thursdays, 7:30–10:00 a.m. on Fridays, and 7:30–11:00 a.m. on Saturdays (Department of Health of Malang, 2012).

##### Participants

Two hundred twenty-two Indonesian adults with T2DM were included in this study. The Rule of 5 from Bryant and Yarnold (1995), used in this study to calculate the sample size, stipulates that the subject-to-variable ratio should not be less than five. The Rule of 200 from Guilford (1954) was also used, which suggests that *N* should be at least 200 cases (Garson, 2008; Shah, 2012). Three inclusion criteria were used to select samples, including being ≥ 20 years old, having a confirmed diagnosis of T2DM, and being willing to participate. Those unable to read and write Indonesian and those with severe diabetes complications such as blindness, amputation, and renal failure were excluded.

##### Procedure

Potential participants were identified by doctors and nurses working in the PHCs and provided with study information sheets and consent forms. When the prospective participant clearly understood the study and agreed to participate, he or she signed the consent form. Data were collected by the first author and the research assistants.

### Data Analysis

On the basis of the results of psychometric testing in the original Taiwanese study (Lin et al., 2008), a CFA using maximum likelihood estimation was performed to test the consistency of the factor structure with the original version. CFA is used to examine the extent to which, a priori, the theoretical model of factor loadings provides an adequate fit for the actual data (Brown & Moore, 2012; Fabrigar et al., 1999). Descriptive analysis and CFA were performed using IBM SPSS AMOS Version 23 (IBM, Inc., Armonk, NY, USA) software. In the CFA, a good-fitting model is deemed to be one that has a weighted chi-square (*x*^2^)/*df* < 3 (Bollen, 1990; MacCallum et al., 1996), a cumulative fit index (CFI) > .90 (Kline, 2005), and a root of mean square error of approximation (RMSEA) < .06 (Browne &Cudeck, 1992; Hooper et al., 2008), with *z* = 0.30 used as a cutoff for items loading onto a factor (Watson & Thompson, 2006). A model was considered to have an adequate fit if two of the above three criteria were met and if the third criterion had an acceptable but not good fit (e.g., RMSEA < .80; Browne & Cudeck, 1992; Hooper et al., 2008). The average variance extracted (AVE) and composite reliability (CR) were calculated to evaluate the construct validity, with the AVE expected to score ≥ .5 and CR expected to score > .7 (Fornell & Larcker, 1981). For reliability testing, the instrument was considered to have acceptable internal reliability when Cronbach's alpha was ≥ .70 for the overall scale and all of the subscales (Pallant, 2010).

Item analysis was performed using SPSS 23.0 (IBM, Inc., Armonk, NY, USA) to determine the continued inclusion or removal of individual items in the instrument. Items that met any two of following criteria were eliminated: (a) The means of the items were either extreme or the variance was zero, (b) items with skewness > 3 or kurtosis > 10, (c) low item discrimination (*SD* < 0.75), (d) the corrected item–total correlation was < 0.3, (e) the Cronbach's alpha of the total scale increased when an item was dropped, and (f) factor loading was < .5 (Lee et al., 2016).

### Ethical Approval

The study was approved by the Health Research Ethics of the National Institute of Health Research and Development, Indonesia Ministry of Health (Reference No. LB.02.01/5.2/KE.513/2013). All of the participants provided written informed consent.

## Results

### Participant Characteristics

Two hundred fifty-six participants were eligible for participation. Ten refused because of lack of sufficient spare time, and 24 declined because of lack of interest. The ages of the remaining 222 participants ranged from 25 to 81 years (mean = 55.2, *SD* = 10.8). Men and women were equally represented, with women (50.9%) holding a slight majority. Most participants were married (87%), nearly one third (29.3%) were university educated, one quarter (24.3%) were unemployed, and most (52.3%) earned a low monthly income (< 74.6 USD). The average duration of having diabetes was 4.3 ± 4.4 years, ranging from 0.02 to 25 years, and most (81.5%) received oral drug treatment. Only 4.5% received regular insulin injections.

### Factorial Construct Validity

**Figure 1:**
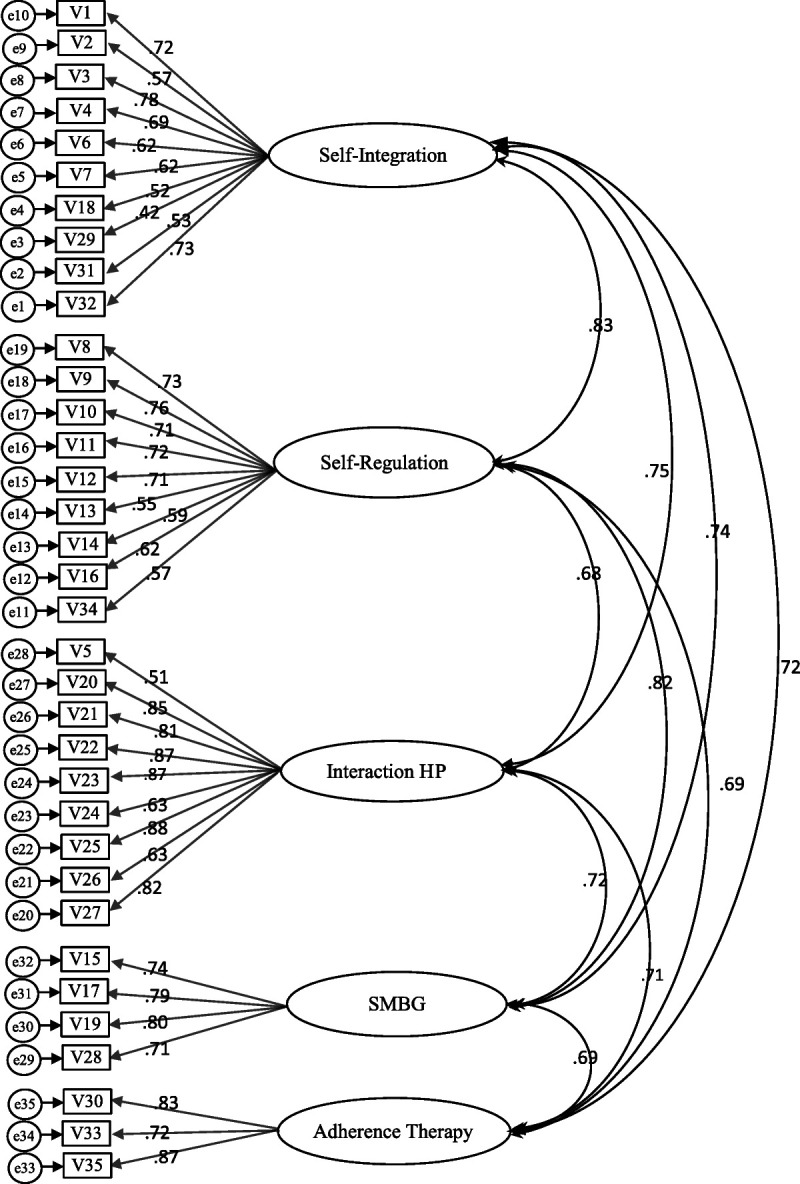
The Indonesian Version of the 35-Item Diabetes Self-Management Instrument Based on the Original Model With Factor Loadings and Interfactor Correlations (*N* = 222) *Note.* HP = health professional; SMBG = self-monitoring blood glucose. Chi-square = 1777.97 (df = 550, *p* < .01), χ^2^/df = 3.2; CFI =.770, RSMEA=.101.

The results of the item analysis are presented in Table 1. No items were deleted based on the item analysis. CFA was used to test construct validity (Watson & Thompson, 2006). The original model for the IDN-DSMI (five domains with 35 items) was identified as an inferior good-fitting model based on two of three criteria ((*x*^2^)/*df* = 3.2, CFI = .770) and an adequate fit using the remaining fit statistic (RMSEA = .101; Figure 1). The raw chi-square was 1777.97 (*df* = 550, *p* < .01). Although the CRs of all the factors were satisfactory (> .7), the AVE of this model was unacceptable (the AVEs of two factors were < .5). Thus, this model was rejected, and further modifications were made.

**Table 1:** Results of Item Analysis

No. Item	Item	Mean	*SD* < 0.75	Kurtosis > 10	Skewness > 3	Corrected Item–Total Correlation	Cronbach's α of the Total Scale Increased When an Item Was Deleted	Factor Loading < .5
Self-integration								
1	Considering the effect on my blood sugars when choosing foods and portions to eat.	2.634	0.979	−1.004	0.060	.609	.958	.665
2	Managing diabetes and participating in social activity.	2.778	1.015	−0.953	−0.321	.532	.958	.569
3	Managing food portions and choices when eating out.	2.781	1.024	−1.050	−0.263	.664	.957	.737
4	Managing diabetes as way to stay healthy	2.963	1.029	−0.881	−0.548	.594	.958	.687
6	Daily life style is healthier than before because of having diabetes.	2.635	0.997	−0.912	−0.221	.536	.958	.598
7	Successfully merged diabetes into daily life.	2.588	1.029	−1.051	−0.106	.577	.958	.615
18	Adjust diabetes routine to fit new situations (such as being away from home, changing my schedule, and celebration).	2.514	1.056	−1.127	−0.060	.551	.958	.561
29	Manage food choices to help control blood glucose.	3.066	0.938	−0.516	−0.663	.719	.957	.733
31	Exercise enough to help control blood glucose and weight.	2.703	0.998	−0.957	−0.195	.377	.959	.406 ^a^
32	Keep weight within the range set up by my healthcare provider and me.	2.785	0.989	−0.791	−0.392	.517	.958	.540
Self-regulation								
8	Pay attention to body signals related to blood glucose level.	2.757	1.008	−1.027	−0.219	.647	.958	.690
9	Pay attention to situations in daily life that may cause blood glucose levels to change.	2.864	0.999	−0.773	−0.481	.670	.957	.723
10	Recognize which signs and symptoms tell the most about blood glucose level.	2.837	1.014	−0.749	−0.521	.611	.958	.677
11	Figure out the reasons for changes in blood glucose levels.	2.620	0.980	−0.824	−0.267	.606	.958	.696
12	Compare the differences between current blood sugar levels and target blood glucose levels.	2.543	1.043	−1.097	−0.032	.603	.958	.703
13	Monitor progress toward desired goals by keeping track of blood glucose levels and A1c.	2.428	1.139	−1.359	0.034	.485	.959	.560
14	Take action based on body signals such as thirst, losing my temper, and feeling anxious.	2.597	0.971	−0.859	−0.130	.528	.958	.595
16	Making decision based on experience	2.704	1.018	−0.996	−0.228	.637	.958	.641
34	Know how to treat if get a low blood glucose	2.917	1.015	−0.806	−0.530	.626	.958	.585
Interaction with health professionals and significant others								
5	Comfortable asking other for tips about managing diabetes.	2.605	0.958	−0.826	−0.131	.610	.958	.524
20	Comfortable asking healthcare provider questions about treatment plan.	3.040	0.944	−0.349	−0.727	.710	.957	.858
21	Work with healthcare providers to identify the possible causes when diabetes control is poor.	2.954	0.944	−0.444	−0.621	.682	.957	.824
22	Comfortable telling healthcare provider how much flexibility in treatment plan.	2.997	0.985	−0.548	−0.676	.711	.957	.853
23	Comfortable telling healthcare provider about changes I would like to make in treatment plan.	2.919	0.981	−0.653	−0.557	.697	.957	.844
24	Tell others about the situations in which need their help for controlling my diabetes.	2.847	0.945	−0.602	−0.462	.614	.958	.628
25	Comfortable discussing the results of out-of-range blood glucose tests with my healthcare providers.	3.063	0.936	−0.354	−0.728	.756	.957	.889
26	Ask others to help with high blood glucose reaction if needed.	2.789	0.987	−0.805	−0.383	.583	.958	.646
27	Comfortable asking healthcare provider about resources that could help manage diabetes.	3.043	0.957	−0.506	−0.685	.744	.957	.827
Self-monitoring blood glucose								
15	Check blood glucose levels when feel as though blood glucose is too low.	2.742	1.077	−1.019	−0.424	.581	.958	.703
17	Check blood glucose when feeling unwell.	2.777	1.044	−1.036	−0.341	.642	.958	.759
19	Check blood glucose level when feeling as though blood glucose is too high.	3.019	0.958	−0.583	−0.627	.760	.957	.866
28	Check blood glucose to help make self-care decisions (e.g., medications, diet, exercise).	3.004	0.924	−0.506	−0.724	.671	.957	.710
Adherence to recommended therapy								
30	Take diabetes medications at the times prescribed.	3.377	0.904	1.115	−1.432	.673	.957	.830
33	See diabetes provider every 1–3 months.	3.119	0.997	−0.356	−0.869	.601	.958	.719
35	Take the amount of diabetes medication that has been prescribed.	3.326	0.860	0.856	−1.242	.650	.958	.875

^a^Represents modulus of skewness > 3, modulus of kurtosis > 10, *SD* < 0.75, item–total correlation < 0.3, and factor loading < .5; Cronbach's alpha increased when item dropped.

^b^Represents the item deleted after item analysis (there were no items deleted from the analysis).

**Figure 2:**
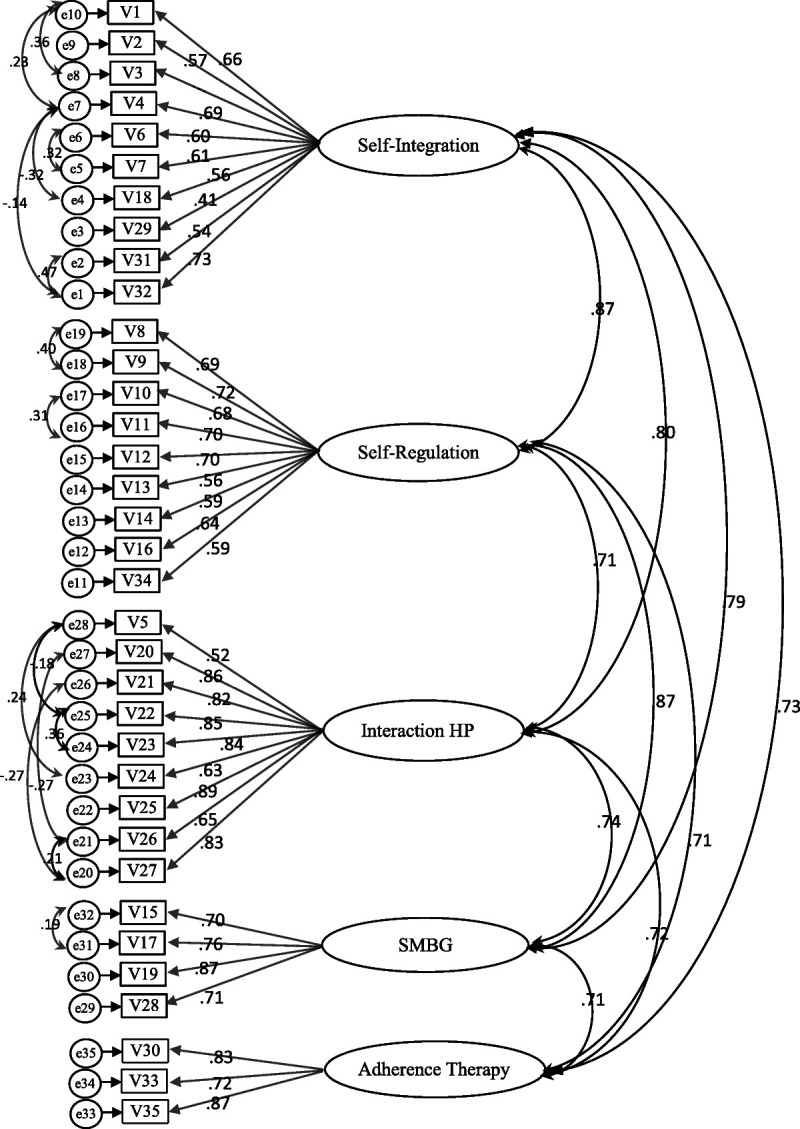
Final Model of the Indonesian Version of the 35-item Diabetes Self-Management Instrument with Factor Loadings, Interfactor Correlations, and Covariance Correlations (*N* = 222) *Note.* HP = health professional; SMBG = self-monitoring blood glucose. Chi-square = 1458.834 (df = 535, *p* < .01), χ^2^/df = 2.727; CFI = .827, RSMEA = .088.

The initial modification, which added the covariance correlations, improved the goodness of fit ((*x*^2^)/*df* = 2.727, CFI = .827, RMSEA = .088), although the AVE remained unchanged (Figure 2). Further sequential modification considering the modification indices, item loading, and residual analysis suggested deleting at least 11 items to achieve a quite good-fitting model ((*x*^2^)/*df* = 2.38, CFI = .911, RMSEA = .079) with acceptable AVE and CR (the AVEs of the constructs were all > .5, and the CRs of the constructs were all > .7). From the 11 items suggested for deletion, five were from the self-integration domain and four were from the self-regulation domain. In the self-integration domain, the items suggested for deletion included questions on managing diabetes in daily life such as “daily lifestyle is healthier than before because of having diabetes,” “successfully merged diabetes into daily life,” “adjust diabetes routine to fit a new situation,” “exercise to control blood glucose,” and “keep weigh within the recommended range.” In the self-regulation domain, the items suggested for deletion included “pay attention to signals of the body related to blood glucose level,” “monitor progress toward desired goals by keeping track of blood glucose levels and A1c,” “decide action based on experience,” and “know how to treat if blood glucose levels become low.” Besides, the two items “comfortable asking other people with diabetes for tips about managing diabetes” and “check blood glucose to help make self-care decisions” were also considered for deletion based on the analysis. However, every modification resulted in an unstable fit. In addition, the items suggested for deletion in the analysis were all considered key points of DSM. Thus, their deletion to achieve a good-fitting model did not make theoretical sense. MacCallum et al. (1992) warned that “when an initial model fits well, it is probably unwise to modify it to achieve an even better fit because modifications may simply be benefitting small, idiosyncratic characteristics of the sample” (p. 501). Besides, using the initial construct must achieve good reliability (the Cronbach's alpha of each scale ranged from .84 to .93). Therefore, the final IDN-DSMI retained all 35 items in the original model.

### Internal Consistency Reliability

The Cronbach's alpha of the final model was .96. The level of internal consistency for each subscale was .86 for self-integration, .87 for self-regulation, .93 for interactions with health professionals and significant others, .86 for self-monitoring blood glucose, and .84 for adherence to recommended therapy (Table 2).

**Table 2:** Factor Loading of IND-DSMI Final Model: AVE, CR, and Cronbach's Alpha

No. Item	Factor	Factor Loading
Factor 1: self-integration (AVE = .38, CR = .86, Cronbach's α = .86)		
1	I consider the effect on my blood sugars when choosing foods and portions to eat.	.665
2	I can participate in the social activities and still manage my diabetes.	.569
3	I know how to manage food portions and choices when I eat out.	.737
4	I regard my diabetes management as a way to stay healthy overall.	.687
6	My daily life style is healthier than before because of having diabetes.	.598
7	I have successfully merged diabetes into my daily life.	.615
18	I can adjust my diabetes routine to fit new situations (such as being away from home, changing my schedule, and celebration).	.561
29	I manage my food choices to help control my blood glucose.	.733
31	I exercise enough to help control my blood glucose and my weight.	.406
32	I keep my weight within the range set up by healthcare provider and me.	.540
Factor 2: self-regulation (AVE = .43, CR = .87, Cronbach's α = .87)		
8	I pay attention to signals my body gives me related to my blood glucose level.	.690
9	I pay attention to situations in my daily life that might cause my blood glucose levels to change.	.723
10	I can recognize which signs and symptoms tell me the most about my blood glucose level.	.677
11	I can usually figure out the reasons for changes in my blood glucose levels.	.696
12	I compare the differences between my current blood sugar levels and my target blood glucose levels.	.703
13	I monitor my progress toward my desired goals by keeping track of blood glucose levels and A1c.	.560
14	I take action based on body signals such as thirst, losing my temper, and feeling anxious.	.595
16	I decide what action to take based on the results of my previous actions.	.641
34	If I get a low blood glucose reaction I know how to treat it.	.585
Factor 3: interaction with health professionals and significant others (AVE = .60, CR = .93, Cronbach's α = .93)		
5	I am comfortable asking other people with diabetes for tips about managing diabetes.	.524
20	I am comfortable asking my healthcare provider questions about my treatment plan.	.858
21	I work with my healthcare providers to identify the possible causes when my diabetes control is poor.	.824
22	I am comfortable telling my healthcare provider how much flexibility I want in my treatment plan.	.853
23	I am comfortable telling my healthcare provider about changes I would like to make in my treatment plan	.844
24	I tell others (e.g., my friends, my family) about the situations in which I need their help for controlling my diabetes.	.628
25	I am comfortable discussing the results of out-of-range blood glucose tests with my healthcare providers.	.889
26	I ask others (e.g., my friends, my family) to help me with my high blood glucose reaction if needed.	.646
27	I am comfortable asking my healthcare provider about resources that could help me manage my diabetes.	.827
Factor 4: self-monitoring blood glucose (AVE = .58, CR = .83, Cronbach's α = .86)		
15	When I feel as though my blood glucose is too low, I check my blood glucose levels as soon as possible.	.703
17	When I feel unwell but I am not sure if the cause is either high or low blood glucose, I check my blood glucose as soon as possible.	.759
19	When I feel as though my blood glucose is too high, I check my blood glucose levels as soon as possible.	.866
28	I check my blood glucose to help me make self-care decisions (e.g., medications, diet, exercise).	.710
Factor 5: adherence to recommended therapy (AVE = .66, CR = .85, Cronbach's α = .84)		
30	I take my diabetes medications at the times prescribed.	.830
33	I see my diabetes provider every 1–3 months.	.719
35	I take the amount diabetes medication that has been prescribed for me.	.875

***Note.*** Cronbach's α of all scales = .96. IND-DSMI = Indonesian-Version Diabetes Self-Management Instrument; AVE = average variance extracted; CR = composite reliability.

### Item Correlations

The examination of item-to-item correlations highlighted that some items were highly correlated (*r* ≥ .70; full item correlation table shown in Table 3). Furthermore, strong interitem correlations were found among Items 20–23 and 25 and among Items 23, 25, and 27, with all of the items in the domain of “interaction with health professionals and significant others” and Items 30 and 35 in the domain of “adherence to recommended therapy.” It also indicated that some items may be redundant.

**Table 3:** Interitem Correlation Matrix of Indonesian-Version Diabetes Self-Management Instrument

No. Item	V1	V2	V3	V4	V9	V10	V11	V12	V14	V15	V17	V19	V20	V21	V22	V23	V24	V25	V26	V27	V29	V30	V33	V35
V1	1.000	.406	.678	.609	.439	.259	.289	.364	.145	.337	.451	.446	.462	.414	.431	.443	.374	.480	.176	.344	.539	.455	.464	.420
V2	.406	1.000	.505	.395	.413	.305	.376	.390	.136	.282	.329	.400	.458	.339	.432	.382	.357	.452	.278	.463	.427	.365	.461	.368
V3	.678	.505	1.000	.598	.494	.403	.398	.474	.299	.316	.396	.496	.461	.395	.432	.390	.335	.499	.288	.437	.546	.394	.445	.437
V4	.609	.395	.598	1.000	.367	.292	.341	.410	.273	.254	.345	.420	.477	.468	.402	.469	.346	.501	.263	.358	.469	.457	.314	.443
V9	.439	.413	.494	.367	1.000	.577	.508	.519	.387	.386	.445	.572	.412	.422	.376	.406	.438	.453	.342	.447	.467	.398	.430	.432
V10	.259	.305	.403	.292	.577	1.000	.634	.487	.541	.424	.403	.494	.364	.409	.400	.345	.329	.389	.383	.450	.328	.414	.317	.437
V11	.289	.376	.398	.341	.508	.634	1.000	.584	.500	.453	.429	.495	.310	.304	.392	.329	.325	.361	.364	.425	.449	.389	.270	.356
V12	.364	.390	.474	.410	.519	.487	.584	1.000	.378	.438	.453	.516	.317	.349	.307	.324	.268	.344	.292	.379	.461	.401	.345	.363
V14	.145	.136	.299	.273	.387	.541	.500	.378	1.000	.487	.384	.511	.269	.318	.336	.364	.327	.335	.432	.407	.295	.347	.279	.359
V15	.337	.282	.316	.254	.386	.424	.453	.438	.487	1.000	.666	.618	.327	.297	.334	.352	.368	.380	.399	.476	.544	.410	.315	.354
V17	.451	.329	.396	.345	.445	.403	.429	.453	.384	.666	1.000	.686	.474	.422	.419	.358	.420	.488	.376	.418	.564	.501	.406	.420
V19	.446	.400	.496	.420	.572	.494	.495	.516	.511	.618	.686	1.000	.564	.482	.556	.509	.553	.523	.431	.557	.585	.540	.543	.527
V20	.462	.458	.461	.477	.412	.364	.310	.317	.269	.327	.474	.564	1.000	.719	.751	.759	.555	.778	.418	.650	.530	.571	.497	.492
V21	.414	.339	.395	.468	.422	.409	.304	.349	.318	.297	.422	.482	.719	1.000	.737	.714	.516	.721	.498	.588	.433	.473	.443	.497
V22	.431	.432	.432	.402	.376	.400	.392	.307	.336	.334	.419	.556	.751	.737	1.000	.827	.510	.743	.527	.681	.474	.467	.452	.461
V23	.443	.382	.390	.469	.406	.345	.329	.324	.364	.352	.358	.509	.759	.714	.827	1.000	.559	.729	.504	**.717**	.453	.473	.395	.440
V24	.374	.357	.335	.346	.438	.329	.325	.268	.327	.368	.420	.553	.555	.516	.510	.559	1.000	.523	.469	.479	.420	.384	.419	.358
V25	.480	.452	.499	.501	.453	.389	.361	.344	.335	.380	.488	.523	.778	.721	.743	.729	.523	1.000	.579	.768	.561	.594	.488	.568
V26	.176	.278	.288	.263	.342	.383	.364	.292	.432	.399	.376	.431	.418	.498	.527	.504	.469	.579	1.000	.683	.398	.383	.369	.403
V27	.344	.463	.437	.358	.447	.450	.425	.379	.407	.476	.418	.557	.650	.588	.681	.717	.479	.768	.683	1.000	.547	.500	.517	.562
V29	.539	.427	.546	.469	.467	.328	.449	.461	.295	.544	.564	.585	.530	.433	.474	.453	.420	.561	.398	.547	1.000	.600	.459	.506
V30	.455	.365	.394	.457	.398	.414	.389	.401	.347	.410	.501	.540	.571	.473	.467	.473	.384	.594	.383	.500	.600	1.000	.533	.738
V33	.464	.461	.445	.314	.430	.317	.270	.345	.279	.315	.406	.543	.497	.443	.452	.395	.419	.488	.369	.517	.459	.533	1.000	.653
V35	.420	.368	.437	.443	.432	.437	.356	.363	.359	.354	.420	.527	.492	.497	.461	.440	.358	.568	.403	.562	.506	.738	.653	1.000

*Note*. Bold values indicate that value were above .7 (high inter-items correlation).

## Discussion

The purpose of this study was to evaluate the validity and reliability of the IDN-DSMI. CFA was used to determine whether the original model may be applied on the IDN-DSMI model as well.

On the basis of a thorough investigation of the literature, this article is believed to be the first study to develop an Indonesian version of the DSMI and to examine its psychometric properties in adults with T2DM in Indonesia. Our findings suggested that IDN-DSMI attained good validity and reliability. The CFA supported acceptable goodness of fit for all of the 35 items, which cover the same five domains as the English version. Furthermore, these findings are consistent with the CFA results conducted in other countries (Taiwan, Iran, and Vietnam; Dao-Tran et al., 2017; Lin et al., 2008; Tol et al., 2011).

In this study, the CFA indicated a need to remove some items. However, doing so would not make sense theoretically and would probably be unwise to make modifications only to achieve better statistical results (MacCallum et al., 1992; Schreiber et al., 2006). Therefore, the final version of the IDN-DSMI retains the same set of items as in the original because the reliability of the original instrument was shown to be excellent. The items with lower loadings and higher residuals may be more sensitive to differences in cultural, education, and social variables across country settings. Compared with countries such as Taiwan and Vietnam, Indonesia has fewer resources and facilitation assistance available to support DSM. Moreover, the health education system in clinical settings, particularly in the primary health services, remains limited in Indonesia, which might be less optimal for the patients. In addition, sampling bias (participants were only recruited from public health services) may have biased the results. In addition, demographic characteristics such as age, level of education, family income, and occupation may have influenced the findings. Although some of the participants had a university degree, the proportion of participants with a less-than-university-degree education was much higher. Participants with lower levels of education tend to prefer that information be presented simply and in a manner that can be easily understood (Baker et al., 2011; Nutbeam, 2008).

In our findings, self-integration was the domain with most items designated for removal. Three of these, including “daily lifestyle is healthier than before because of having diabetes,” “successfully merged diabetes into daily life,” and “adjust diabetes routine to fit a new situation,” conveyed similar contents and may be redundant. In addition, two items, including “exercise to control blood glucose” and “keep body weight within the recommended range,” may relate to Indonesians with low self-awareness to do exercise and keep a healthy body weight. Thus, these two items had the lowest factor loading. In the self-regulation domain, the items designated for removal were related to decision making, which may be influenced by the level of knowledge, such as “pay attention to signals of the body related to blood glucose level,” “decide action based on experience,” and “know how to treat low blood glucose.” The item “monitor progress toward desired goals by keeping track of blood glucose levels and A1c” was also designated for removal, perhaps because participants were unfamiliar with using A1c as a monitoring parameter of blood glucose level. Diabetes testing in this study was conducted primarily in PHC settings, which did not have the facilities necessary to measure HbA1c. Although HbA1c is one of the international standards for measuring DSM, most healthcare facilities in Indonesia, especially primary care settings, do not have the tools necessary to measure this variable. Thus, traditional tools such as the blood glucose stick are still widely used to monitor blood glucose levels.

The IDN-DSMI achieved the preferred internal consistency (α = .96), which is comparable with the instrument validations conducted in Taiwan (α = .94), Iran (α = .91), and Vietnam (α = .92; Dao-Tran et al., 2017; Lin et al., 2008; Tol et al., 2011). This evaluation suggests that IDN-DSMI is a reliable tool for measuring the concept of DSM among Indonesians with diabetes. However, a Cronbach's alpha of .90 or higher indicates the possibility of unnecessary items (Tavakol & Dennick, 2011). Besides, the high item-to-item correlation suggests that most of the questions overlap. Thus, further study may beneficial to investigate the potential for developing a shorter version of the IDN-DSMI.

This study also indicates that the problem of poor-fitting model may relate to limitations inherent to the healthcare system, particularly primary healthcare, and infrastructure in Indonesia. Promoting the health education competence of PHC medical personnel is essential to supporting patients with diabetes. Providing psychosocially based educational interventions and addressing cultural issues that may improve patients' self-care behavior are also essential (Tan et al., 2018). Moreover, providing an empowerment program to people with diabetes may be beneficial to improving DSM (Chen et al., 2017).

### Limitations

First, the instrument was validated in adults with T2DM in the outpatient department of PHCs in Malang City, Indonesia, only. Therefore, this study may not be generalizable to other populations. Second, the demographic characteristics of the participants, particularly in terms of level of education, was quite extreme (nearly three quarters with less than a university degree). Thus, future investigations should better reflect the demographic characteristics of the general population by sampling a broader population of patients.

### Conclusions

The IDN-DSMI is a valid and reliable instrument for measuring DSM behavior in the Indonesian community, especially among patients in primary healthcare. Cultural factors and facilities supporting healthcare services may cause problems of poor fit model. The findings highlight the importance of promoting the health education system and improving infrastructures to promote better DSM by patients with diabetes.

### Relevance to Clinical Practice

The IDN-DSMI is a new tool for assessing the self-management behavior of patients with diabetes. This tool may be used by healthcare providers to identify patient problems relating to DSM.
